# The Reliability and Relevance of a Quality of Decision Making Instrument, Quality of Decision-Making Orientation Scheme (QoDoS), for Use During the Lifecycle of Medicines

**DOI:** 10.3389/fphar.2019.00017

**Published:** 2019-01-23

**Authors:** Magdalena Bujar, Neil McAuslane, Stuart Walker, Sam Salek

**Affiliations:** ^1^Centre for Innovation in Regulatory Science, London, United Kingdom; ^2^Department of Pharmacy, Pharmacology and Postgraduate Medicine, School of Life and Medical Sciences, University of Hertfordshire, Hatfield, United Kingdom

**Keywords:** measurement instrument, validation, R&D, pharmaceutical, regulatory, health technology assessment

## Abstract

**Introduction:** The Quality of Decision-Making Orientation Scheme (QoDoS) was developed to provide organisations involved in submission, approval and reimbursement of new medicines with a tool to improve the quality of their decision-making processes and is considered the most promising tool for such purpose. This study aimed to further establish the measurement properties of the QoDoS by evaluating its reliability (internal consistency and test-retest reliability) and relevance in the target population.

**Methods:** The study participants consisted of 55 individuals recruited from pharmaceutical companies, regulatory and HTA agencies. It was designed as a longitudinal study with participants assessed on two different occasions, at baseline (test 1) and then 7 days later (test 2). Internal consistency reliability was assessed with Cronbach’s alpha and the test-retest reliability was evaluated using the intraclass correlation coefficients (ICC) based on absolute agreement, 2 way mixed-effects model for the four QoDoS domains. The relevance of the QoDoS was evaluated by applying cognitive debriefing using five short feedback questions following test 1.

**Results:** Test 1 was completed by 44 study participants (80% response rate) and test 2 was completed by 32 of the 44 individuals, resulting in a 73% response rate. Cronbach’s alpha coefficient was greater than 0.7 across all the domains for test 1 and test 2, ranging from 0.71 to 0.79, indicating good consistency of responses. For the overall score across all 47 items, the Cronbach’s alpha coefficient was 0.81 for test 1 and 0.86 for test 2, which is rated as very good. The four QoDoS domains showed moderate to strong reproducibility (ICC range: 0.63–0.86). The outcome of the cognitive debriefing from the 43 respondents (98% response rate) confirmed the relevance (95% agreement), language clarity (95%) and completeness of items (86%); the clarity of the scaling (91%) as well as spontaneity of responses (95%).

**Conclusion:** These results provide strong support for the relevance and reliability of the QoDoS, which are key properties for future longitudinal and cross-sectional applications of the instrument when evaluating quality of decision making by those involved in the lifecycle of medicines.

## Introduction

In the absence of a validated instrument for measuring quality of the decision-making processes throughout the lifecycle of medicines, the QoDoS was developed using a standardised, established approach for the design and evaluation of such measures. The QoDoS possesses certain psychometric properties, which have already been demonstrated by Donelan et al. (2016), namely face validity (instrument assesses desired qualities), content validity (instrument includes a representative set of items) and construct validity (the results obtained from the use of a measure fit the theoretical foundations from which it is designed) (Trochim, 2006; Streiner et al., 2015). In addition, the QoDoS is easy to understand and can be completed in a short time frame (Donelan et al., 2015, 2016). The practicality of the tool in a regulatory authority and pharmaceutical company setting was confirmed through a study with 76 participants (50% from regulatory authorities and 50% from pharmaceutical companies) (Bujar et al., 2016). The findings of this pilot study as well as the results of a recent systematic review (Bujar et al., 2017) have demonstrated that the QoDoS is considered to be the most promising instrument for evaluating quality of the decision-making process during medicines development and review, identifying strengths and weaknesses and raising awareness of the issues in quality decision making across individuals and within organisations. The challenge is how to ensure that the QoDoS, in addition to the above described attributes, produces reliable, and relevant results.

Although internal consistency reliability of the QoDoS had already been carried out (Donelan et al., 2016), which has demonstrated the relatedness/homogeneity of the tool, external reliability testing to determine that the QoDoS produces stable results has not been undertaken as part of the initial tool development nor has the relevance of the final tool been demonstrated in the target audience. This was due to resource and timing constraints, but recognising the importance of those properties, it has been carried out as a follow-on study described in this paper. It is hoped that the additional reliability and validity testing undertaken here have further strengthened the measurement properties of the QoDoS to increase its acceptability as a potential gold standard for evaluating quality of decision making during the lifecycle of medicines.

The relevance of an instrument can be evaluated by applying cognitive debriefing, a technique of actively testing the tool among representatives of the target population. The aim would be to determine the perception of the participants regarding the relevance, language clarity and completeness of the QoDoS items (Brod et al., 2009; Streiner et al., 2015). Reliability (overall consistency of a measure), on the other hand, reflects the scale’s ability to produce similar results under consistent conditions. It can be demonstrated in two ways, firstly through internal consistency, based on a single administration of the measure, which represents the average of the correlations among all the items in the measure (Streiner et al., 2015). Cronbach’s alpha determinations have already been applied to measure this by Donelan et al. (2016) and the QoDoS showed high internal consistency (*n* = 120, Cronbach’s alpha = 0.89), which was re-examined through this study. Secondly, where a measurement technique is used over time, reliability can be determined by the reproducibility of the scores on different occasions. This can be demonstrated with test-retest reliability by evaluating whether an instrument yields the same scores over time with multiple administrations, assuming subject stability (Streiner et al., 2015), namely that the decision-making practises of the individuals and the perception of their organisation has not changed during the test period.

The aim of this study was to further examine the psychometric properties of the QoDoS tool in the target audience, namely pharmaceutical companies, regulatory authorities, and HTA agencies. The objectives across the three stakeholders were to:

1. Confirm the internal consistency of the scores of the four domains and the summary score of the QoDoS.
2. Assess the test-retest reliability of the scores of the four domains and the summary score of the QoDoS.
3. Apply cognitive debriefing to evaluate the relevance, language clarity and completeness of the QoDoS items; the clarity of the scaling as well as spontaneity of responding to the questionnaire.

## Materials and Methods

### Design of the Study

This study was designed as a longitudinal study, with participants assessed on two different occasions (test 1 and test 2) with a 7-day interval. A period of 7 days has been recommended to minimise bias and to avoid under- or over-estimation of the test scores (Streiner et al., 2015). The aim was therefore to ensure that the condition of the participants remained stable (Paiva et al., 2014). Internal consistency reliability of the responses was determined for both test 1 and test 2. In addition, the relevance of the content of the QoDoS to the target population was evaluated.

#### Ethics Committee Permission

The study protocol received approval from the University of Hertfordshire Institutional Ethics Committee. Since the study participants were neither National Health Service patients or staff, it did not require the Local Ethics Committee approval. The recruited participants received a copy of the study protocol describing the purpose of the study and explaining that only aggregated results will be reported prior to their participation. Therefore, their agreement to participate in the study constituted consent.

### Assessment Tool: The Quality of Decision-Making Orientation Scheme (QoDoS)

The 47-item QoDoS (Appendix 1) has two parts, where Part 1 relates to the Organisation, consisting of two domains (“Approach,” items 1–12; and “Culture,” items 13–23) and Part 2 relates to the Individual, with two domains (“Competence,” items 24–37; and “Style,” items 38–47). As many decisions are made by individuals every day, the participants were asked to complete the instrument relating to their views on their personal and their organisation’s decision-making processes for major strategic choices within their organisation.

The 47 QoDoS items were rated as either favourable or unfavourable using an expert panel (Table 1); for example, item 2 “My organisation’s decision making is transparent” represents a favourable practise, whereas item 13 “My organisation has suffered a negative outcome due to slow decision making” represents unfavourable practise. Based on this, the Likert scale response options were quantified by assigning scores to each of the response scale. For QoDoS items considered as favourable practise, the following scores were assigned where Not at all = 0, Sometimes = 1, Frequently = 2, Often = 3, and Always = 4. For QoDoS items considered as unfavourable practise, the reverse scores were assigned where Not at all = 4, Sometimes = 3, Frequently = 2, Often = 1, and Always = 0 (Table 1). In addition, four background questions were used to collect data on gender, job title, professional experience, and organisation type.

**Table 1:** (QoDoS) items assigned as either favourable or unfavourable practise.

Assignment	QoDoS item number
Favourable practise	1, 2, 3, 6, 7, 8, 9, 10, 11, 12, 22, 23, 24, 25, 26, 27, 28, 29, 30, 31, 32, 33, 34, 35, and 37
Unfavourable practise	5, 13, 14, 15, 16, 17, 18, 19, 20, 21, 36, 38, 39, 40, 41, 42, 43, 45, 46, and 47


### Cognitive Debriefing Questionnaire

In addition, following completion of test 1, the study participants were asked to complete five questions to assess the attributes such as: relevance, where each item should reflect an aspect of importance regarding decision making to the target population; language clarity, where sentences should be clear, understandable, straightforward and simple; scaling, where the format of the categories must be clear and fit with the items and the construct; completeness to ensure that no items are believed to be missing or perceived as repetitive and spontaneity to ensure that the QoDoS can be completed efficiently without prompting or rethinking, thereby maximising response rate and minimising errors or undesirable response behaviour. The following questions were developed for the purpose of this study (Brod et al., 2009; Streiner et al., 2015):

1. Question 1: Did you find the QoDoS items relevant? (yes/no) If no, please specify item number.
2. Question 2: Did you find the QoDoS items easy to understand? (yes/no) If no, please specify item number.
3. Question 3: Did you find the response options easy to understand? (yes/no) If no, please specify.
4. Question 4: Were you able to respond to the QoDoS spontaneously? (yes/no)
5. Question 5: Any items that you believe should be deleted or added (yes/no) If yes, please specify.

### Study Participants

The study participants were recruited from an international workshop on quality decision making (Centre for Innovation in Regulatory Science [CIRS], 2017). These were recruited based on experience using purposive sampling, from those holding senior positions with at least 5 years of experience in a managerial position within major international pharmaceutical companies, regulatory authorities and HTA agencies as well as relevant academic institutions. A sample size of at least 30 individuals for test 1 and test 2 was required (Paiva et al., 2014).

### Study Procedure

Participants were invited in May 2017, with the study planned to take place in June 2017. Prior to the test 1, participants were subject to a 1-day training course on quality decision making in order to ensure that their baseline knowledge of decision making was the same and to minimise learning effect occurring between the initial and second completion of the questionnaire. As a result, it was assumed that the decision-making practises of the individuals and the perception of their organisation had not changed and therefore the participants’ circumstances remained stable. Following completion of the first assessment, participants received a second copy of the QoDoS with a unique identification number, contact information and a note with the completion date indicated (7 days from the initial assessment). They were also informed that they would receive a reminder for the follow-up questionnaire. On the sixth day following their initial assessment, the participants were sent a reminder email regarding completion of the retest on the following day (i.e., the seventh day) together with an electronic copy of the QoDoS for completion. All the respondents who completed tests 1 and 2 with a 7- or an 8-day interval were included in the analysis.

### Data Processing and Analysis

Information was collected manually into an Excel database for the completed questionnaires and subsequently cleaned and coded. The questionnaires were paired based on the unique identification numbers assigned to each copy of the instrument. The analysis was carried out on the 47 QoDoS items using Excel and the Statistical Package for the Social Sciences (SPSS) version 23. Initially, the responses were plotted as a box-and-whisker graph, which indicates the 25 and 75th percentile (box), the 5 and 95th percentile (whiskers) as well as the median (diamond) in order to explore variance within each item as well as when comparing test 1 with test 2. Internal consistency of the QoDoS was assessed using the Cronbach’s alpha coefficient for the inter-item correlation, where a value of 0.7–0.9 was required based on the number of items in the instrument. A value that is too low signifies that some items are not representative, whereas a value that is too high may reflect redundancy amongst items (Streiner et al., 2015). Test-retest reliability was assessed using the ICC, which shows absolute agreement between two scores where a value of >0.7 was required. The ICC also accounts for systematic error and is based on analysis of variance in scores (Paiva et al., 2014; Streiner et al., 2015). For the purpose of this study where the rater was the same (intra-rater reliability) a two-way mixed effects model was chosen and the definition of relationship was considered as absolute agreement (Koo and Li, 2016). Responses to the five questions regarding the relevance were analysed using Excel and the comments were codified and combined where applicable. Due to confidentiality reasons, only aggregated results are shown and no data that identify an individual or a specific organisation were reported.

## Results

This study focused on relevance and reliability testing (internal consistency and test-retest reliability) of the QoDoS in the target population, namely individuals involved in medicines development, regulatory review and HTA of new medicines. For the purpose of clarity, the key results are presented in three parts:

1. Part I – Internal consistency reliability.
2. Part II – Test-retest reliability.
3. Part II – Relevance testing.

### Characteristics of the Study Participants

The initial test (test 1) was completed by 44 individuals from 55 contacted (80% response rate), where 24 (55%) were from pharmaceutical companies, 12 (27%) from regulatory authorities, three (7%) from HTA agencies and five (11%) from a range of relevant academic institutions. In terms of gender, 20 (45%) were male, 22 (50%) female and two individuals did not specify (5%). The individuals had a median of 21 years of work experience, with a range of 5 to 38 years and titles ranging from manager to organisational head and professor in the case of academia. The retest (test 2) was completed by 32 out of the 44 individuals who had completed test 1, resulting in a 73% response rate. In this case, 13 individuals were from companies (41%), 11 from regulatory authorities (34%), three (9%) from HTA agencies and five (16%) from academia. In terms of gender, 15 were male (47%) and 17 female (53%), with a median of 18 years of professional experience ranging from 7 to 38 years and titles varying from manager to organisational head and professor in the case of academia.

### Part I – Internal Consistency Reliability

#### Variance in QoDoS Scores

**FIGURE 1:**
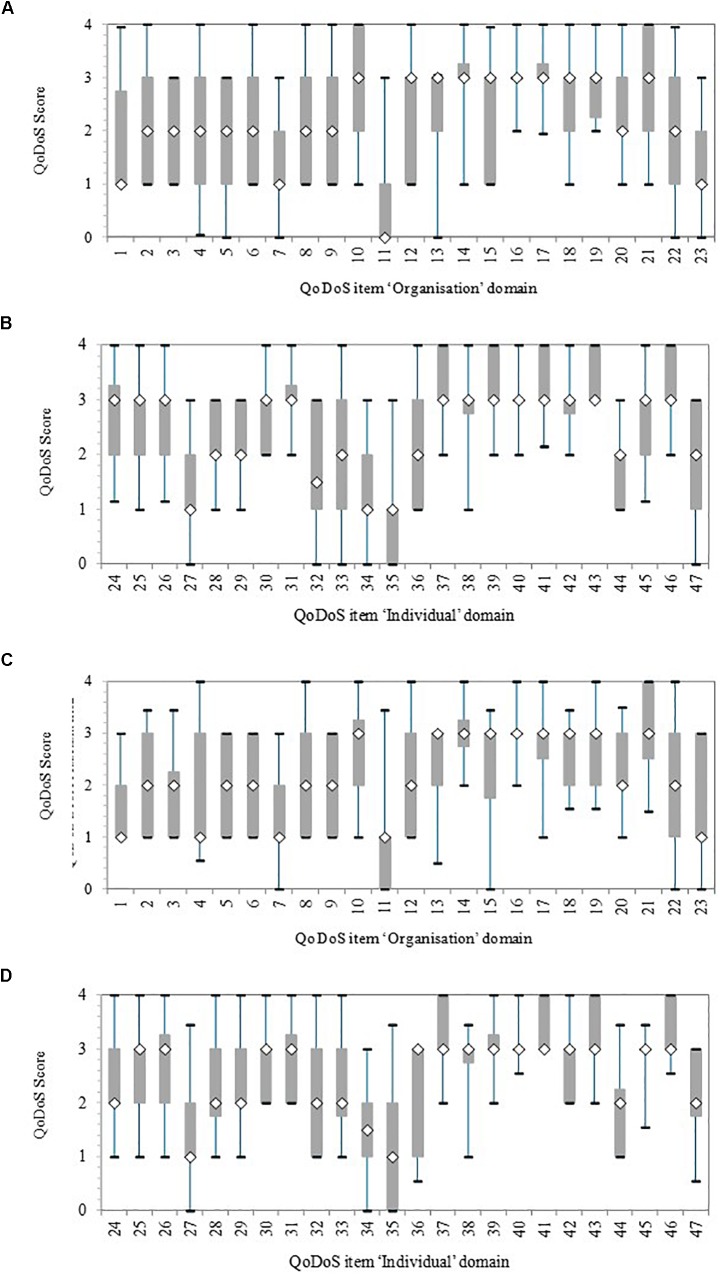
Quality of Decision-Making Orientation Scheme (QoDoS) scores, where whiskers indicate 95 and 5th percentile, the 75 and 25th percentile and the white diamond is the median. Test 1, *n* = 44; Test 2, *n* = 32. **(A)** Test 1 Organisational: Approach (items 1–12) and Culture (items 13–23). **(B)** Test 1 Individual: Competence (items 24–37) and Style (items 38–47). **(C)** Test 2 Organisational: Approach (items 1–12) and Culture (items 13–23). **(D)** Test 2 Individual: Competence (items 24–37) and Style (items 38–47).

Initially, the variance within each item for test 1 and test 2 was illustrated with a box-and whisker plot for the organisational and individual QoDoS parts (Figure 1). The variance was reported in terms of 5, 25, 50, 75, and 95th percentiles. Overall, each item had a considerable variance around the median, generally a difference of two points in the 25 and 75th percentile box.

For test 1, the organisational items with smallest variance in terms of the length of the 25 and 75th percentile box were items 14 (“My organisation’s culture has resulted in its inability to make a decision”), 16 (“My organisation’s decision making results in making the same mistake as in the past”), and 17 (“My organisation’s decision making is influenced by the vested interest of individuals – e.g., conflict of interest”). The items with the biggest variance were 4 (“My organisation uses a structured approach in its decision making”) and 22 (“My organisation effectively communicates the decisions it makes”) according to the largest difference between the 5 and 95th percentiles (whiskers). The test 1 individual items with the smallest variance were items 31 (“I understand the importance of the decisions I make”), 38 (“Emotion is part of my decision making”), 40 (“I have experienced a negative outcome by a decision not being made”) and 42 (“Recent or dramatic events greatly impact my decision making”), whereas the largest variance was for item 33 (“I assign qualitative values to its decision-making criteria”). In general, these differences were also reflected in test 2, although the variance was smaller for a number of items in test 2 compared with test 1, due to smaller sample size.

#### Cronbach’s Alpha Coefficient

Internal consistency of the QoDoS scores was assessed using the Cronbach’s alpha coefficient both for test 1 and test 2 across the pooled sample (Table 2). The assessment was carried out for each of the four QoDoS domains, namely organisational decision-making approach and culture as well as individual decision-making competence and style. Cronbach’s alpha coefficient was greater than 0.7 across all the QoDoS domains for test 1 and test 2, ranging from 0.71 to 0.79, indicating “good” consistency (Streiner et al., 2015). For the overall score across all 47 items, the coefficient was 0.81 for test 1 and 0.86 for test 2, which is considered as very good (Streiner et al., 2015).

**Table 2:** Internal consistency Cronbach’s alpha coefficient for (QoDoS) domains; *n* = number of participants.

	Test 1	Test 2
QoDoS domain	(*n* = 44)	(*n* = 32)
Organisational decision-making approach (12 items)	0.77	0.79
Organisational decision-making culture (11 items)	0.72	0.79
Individual decision-making competence (14 items)	0.76	0.85
Individual decision-making style (10 items)	0.71	0.70
Overall (47 items)	0.81	0.86


### Part II: Test-Retest Reliability

The external consistency (reproducibility) of the QoDoS scores for two completions was assessed. For test 2, 32 individuals returned their responses out of the 44 included in test 1 (73%). Interestingly, from the 12 individuals who did not provide responses to the retest, 11 were from pharmaceutical companies. ICC estimates and their 95% confidence intervals were calculated based on absolute agreement, 2-way mixed-effects model. The four QoDoS domains showed moderate-to-strong reproducibility (ICC range 0.63–0.86). The ICC was lower for the two individual domains (0.63 for competence and 0.72 for style) compared with the two organisational domains (0.86 for approach and 0.82 for culture) (Table 3).

**Table 3:** Test-retest reliability of (QoDoS) domains (*n* = 32).

QoDoS domain	ICC	95% CI		Significance
		Lower bound	Upper bound	
Organisational decision-making approach (12 items)	0.86	0.72	0.93	0.0001
Organisational decision-making culture (11 items)	0.82	0.67	0.91	0.0001
Individual decision-making competence (14 items)	0.72	0.50	0.85	0.0001
Individual decision-making style (10 items)	0.63	0.36	0.81	0.0001


### Part III: Relevance Testing

**FIGURE 2:**
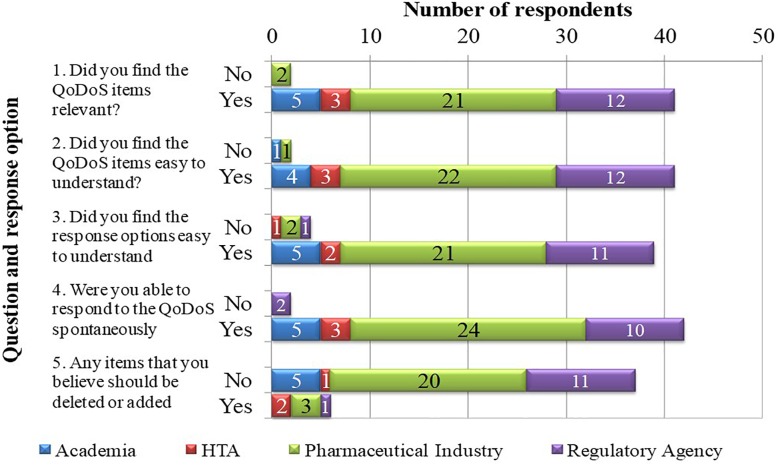
Relevance of the (QoDoS) using cognitive debriefing.

Cognitive debriefing was applied to test the relevance of the QoDoS questionnaire. The five feedback questions included in test 1 of the study were completed by 43 out of the 44 participants, where one individual from a pharmaceutical company did not provide responses (Figure 2). Overall, 41 out of 43 (95%) of the participants considered the QoDoS items relevant, easy to understand and spontaneous to answer based on responses to questions 1, 2, and 4, respectively. For question 3 regarding the response options, 91% of individuals agreed that the response options are easy to understand, whereas 86% of the participants believed the QoDoS tool to be complete and that that no additional items should be added or deleted. The comments provided by the diverging individuals are summarised in Table 4.

**Table 4:** Comments from participants regarding cognitive debriefing to establish (QoDoS) relevance.

Question	Comment
(1) Did you find the QoDoS items relevant (yes/no)? If no please specify item number.	• Don’t think all the items should be equally weighed (*n* = 1) • Many questions were repetitive (*n* = 3)
(2) Did you find the QoDoS items easy to understand? (yes/no) If not please specify item number (s).	• Need to clarify context further (i.e., development process) (*n* = 1) • Unclear whether to respond as an individual versus for the organisation (*n* = 1) • Item 6: my organisation assigns qualitative values to its decision-making criteria (*n* = 1)
(3) Did you find the response options easy to understand? (yes/no) If no please specify.	• Need to differentiate between frequently/often (*n* = 3) • Could benefit from yes/no instead of current answers (*n* = 2)
(4) Any items that you believe should be deleted or added? (yes/no) If yes please specify.	• Availability of tools to support decision making (*n* = 1) • Instructions why tool is required (*n* = 1)


*n = number of participants.*

## Discussion

A recent systematic review has demonstrated that the QoDoS is considered to be the most promising technique for assessing decision making in the lifecycle of medicines (Bujar et al., 2017). The overall benefit of systematically assessing the quality of decision making with the QoDoS is not only to enable an increased awareness of biases and best practises, but also to facilitate measurement of change over time in order to determine the impact of any improvement initiatives. One of the recommendations of the recent systematic review was, therefore, to further test the reliability of the QoDoS. Consequently, this study has demonstrated the relevance and reliability (internal consistency and test-retest reliability) of the QoDoS and thereby provides confidence of its robustness in evaluating quality of decision making during the development and review of medicines.

The variance around the QoDoS scores, particularly for test 1 and also for test 2, was considerable, which reflects the ability of the tool to differentiate between participants regarding their perception of their own decision making as well as that of their organisation. The items with the smallest variance, such as 16 (“My organisation’s culture has resulted in its inability to make a decision”) or 40 (“I have experienced a negative outcome by a decision not being made”), should also be further evaluated in future QoDoS studies. Perhaps for these two items, both referring to timeliness, the scores might have been narrow and generally positive due to the fact that companies and agencies have to make key decisions within a specified time limit (either legislated timelines by regulatory authorities/HTA agencies or business decisions dictated by companies). The rationale and differences in the items with the largest variance should also be explored in future studies with organisations.

The reliability of the QoDoS was assessed for the total scale as well as the four domains (organisational decision-making approach; organisational decision-making culture; individual decision-making competence and individual decision-making style). The results confirmed acceptable internal consistency according to Cronbach’s alpha for the overall instrument and the domains, as compared with the internal consistency of QoDoS evaluated by Donelan et al. (2016). This demonstrates that the domains and the overall instrument are well defined and homogenous in terms of delving into the appropriate aspects of the same construct (quality decision making).

The reproducibility of the QoDoS was also established using test-retest reliability with a 7-day interval between the two assessments. Interestingly, almost all the participants who completed test 1 but not test 2 were from pharmaceutical companies, which suggests that individuals from industry may not recognise the importance of such an exercise or that they are less accustomed to being engaged in these kinds of studies compared with agencies and academia. The results from the test-retest demonstrate a strong level of agreement between the initial and second assessment across the four domains and for the overall QoDoS score. The ICC was lower for the two individual domains of the QoDoS compared with those of the organisation. This may be due to the fact that the perception of an individual regarding their own decision-making abilities may be subject to mood changes or personal circumstances at the time of completion, as opposed to being more objective regarding their assessment of their organisation. Another explanation may be that individuals might have adjusted their decision making during the 7 days following the training session received on the day of the test 1 completion. Nevertheless, the ICC scores for QoDoS domains were all greater than 0.6, indicating that the QoDoS generates results that are precise and objective despite the subjective nature of the topic the scale assesses.

The sample used in this study could be considered small, however, it should be noted that this sample size was acceptable for testing each of the four QoDoS domains, where each domain consists of 10–14 items, which is in line with previous studies reported by Paiva et al. (2014) and the general consensus among the psychometricians (Shoukri et al., 2004) recommending two to three subjects per item.

This study has also demonstrated the relevance of the QoDoS to the target participants, where the feedback from the respondents confirmed the relevance, language clarity and completeness of the QoDoS items and the clarity of the scaling as well as spontaneity to the response process. The comments received by the individuals have also been analysed. Regarding question 1 (relevance of items), individuals commented that items should be equally weighed and that questions were repetitive. Nevertheless, it should be noted that QoDoS items have already been reduced using factor analysis to enable mean completion time of 10 min, whereas item deletion may result in loss of information regarding certain key areas of the construct (Donelan et al., 2016).

Regarding the clarity of the items (question 2), individuals provided comments regarding the need to clarify the decision context beyond just stating “key strategic decisions” Nevertheless, this was not possible for the purpose of this study due to the wide range of participants and their respective decisions, but the need for clarity will be considered in future studies with similar organisations or individuals. In addition, item 6 (“My organisation assigns qualitative values to its decision-making criteria”) was highlighted as unclear and this should be explored further, though noting that only one respondent highlighted this issue. Finally, the lack of clarity regarding the distinction between organisational- and individual-level questions was noted and this could be addressed by making the difference between the QoDoS tables for part 1 and part 2 more apparent (see Appendix 1).

For the response options (question 3), individuals reflected that they did not see the difference between the options “frequently” and “often.” The weighing of the response options was clearly defined in the instrument, that is “Assume that Not at all = 0% of time; Sometimes = 25% of time; Frequently = 50% of time; Often = 75% of time; Always = 100% of time.” However, the definitions could potentially be further highlighted in the questionnaire or the percentages noted in the actual table containing the 47 questions. In addition, two individuals recommended changing the options to “yes” and “no,” but this may result in loss of information, where intermediate options may be required by respondents.

Finally, in question 5 regarding completeness of the tool, participants highlighted that an item could be added regarding the “availability of tools to support decision making” as well as adding “instructions as to why the tool is required.” Whilst the QoDoS already provides insight into the aspects of tool availability to support decision making through item 27, this suggestion could be addressed further by revising this item (“I generate a SWOT analysis in my decision making”) to (“I utilise decision-making tools such as SWOT analysis in my decision making”). The recommendation to provide a rationale for the use of the tool could be addressed by creating a supplementary questions-and-answers document for future QoDoS studies.

The feedback received will be considered during future QoDoS studies in order to further ensure the objectivity and precision of the results obtained. Future studies would also concentrate on establishing the differences between the reliability of the QoDoS across the three groups, pharmaceutical companies, regulatory authorities and HTA agencies, which would require a larger sample. In conclusion, the results of this study provide a strong support for the relevance and reliability of the QoDoS for longitudinal and cross-sectional application of the instrument when evaluating quality of decision making across participants involved in the research and development of medicines.

## Conclusion

This study marks a milestone in validating the QoDoS, an instrument for assessing quality of decision making during medicines development and review. Using well-defined methods and techniques, the results have demonstrated the reliability and relevance of the QoDoS, which are key properties for future application of the instrument in evaluating pharmaceutical companies, regulatory authorities and HTA agencies. These two attributes are crucial for any future applications of the instrument, particularly longitudinal studies in order to ensure that a potential change in decision-making practises is a result of modified organisational processes, as opposed to being due to measurement error. The next step is to apply the QoDoS in pharmaceutical companies, regulatory authorities and HTA agencies, in order to determine the factors that influence decision making within organisations, including favourable practises and those that may require improvement, as well as to identify common themes in quality decision making across the organisations.

## Author Contributions

MB conceived the study, participated in the development of the method, carried out the analysis, and drafted the manuscript. NM, SW, and SS participated in the study design, the development of the method, the design/presentation of results, tables, and reviewed the manuscript.

## Conflict of Interest Statement

SW and SS have joint copyright of the QoDoS tool. The remaining authors declare that the research was conducted in the absence of any commercial or financial relationships that could be construed as a potential conflict of interest.

## Abbreviations

HTA: health technology assessment
ICC: Intraclass Correlation Coefficient
QoDoS: Quality of Decision-Making Orientation Scheme
SWOT: Strengths-Weaknesses-Opportunities-Threats
