# Supplementary material for: The Reliability and Relevance of a Quality of Decision Making Instrument, Quality of Decision-Making Orientation Scheme (QoDoS), for Use During the Lifecycle of Medicines
Source: Front Pharmacol. 2019 Jan 23;10:17. doi: 10.3389/fphar.2019.00017 (PMC6351444; doi:10.3389/fphar.2019.00017)
Supplement: Supplementary file 1 [file Data_Sheet_1.PDF]

Appendix 1: The Quality of Decision-Making Orientation Scheme (QoDoS)

The Quality of Decision-Making Orientation Scheme (QoDoS) ©

The statements in the questionnaire relate to your views on your personal and your organisation's *decision-making processes for major strategic choices within your organisation.*

Please mark clearly one box for each statement. Assume that Not at all = 0% of time; Sometimes = 25% of time; Frequently = 50% of time; Often = 75% of time; Always = 100% of time. If not sure, please tick the box that you feel is the most appropriate.

No data that will identify an individual or an organisation will be reported, or details made to a third party.

Background questions

Gender: ☐ Male ☐ Female ☐ Other

Job title: ____

How many years of professional experience have you to date? ____________________

Organisation: ☐ Regulatory Agency ☐ Pharmaceutical Industry ☐ HTA ☐ Academia ☐ Other

Part I: Organisational-level influences

	Not at all	Sometimes	Frequently	Often	Always	Not applicable
**A. Decision-Making Approach**						
1. My organisation evaluates the impact of the decisions it makes						
2. My organisation's decision making is transparent						
3. My organisation's decision making is consistent						
4. My organisation uses a structured approach in its decision making						
5. My organisation's decision making is influenced by external stakeholder's demands						
6. My organisation assigns qualitative values to its decision-making criteria						
7. My organisation assigns quantitative values to its decision-making criteria						
8. My organisation is open to using better alternatives in its decision making						
9. My organisation encourages innovative decision making						
10. My organisation considers uncertainties in relation to its decision making						
11. My organisation provides training in the science of decision making						
12. My organisation re-examines its decision making as new information becomes available						
**B. Decision-making culture**						
13. My organisation has suffered a negative outcome due to slow decision making						
14. My organisation's culture has resulted in its inability to make a decision						
15. My organisation's decision making is influenced by organisational politics						
16. My organisation's decision making results in making the same mistake as in the past						
17. My organisation's decision making is influenced by the vested interest of individuals (e.g. conflict of interest)						
18. My organisation underestimates problems which adversely impact its own decisions						
19. My organisation continues with projects/products which should be terminated at an earlier stage						
20. My organisation's decision making is influenced by similar organisations or competitors						
21. My organisation's decision making is influenced by incentives or penalty payments						
22. My organisation effectively communicates the decisions it makes						
23. My organisation provides clear and unambiguous instructions for decision making						

© 2017 M.S. Salek, S.R. Walker & R. Donelan

Part II: Individual-level influences

	Not at all	Sometimes	Frequently	Often	Always	Not applicable
**A. Decision-making competence**						
24. My decision making is knowledge based						
25. My decision making is consistent						
26. I consider uncertainty and unknowns in my decision-making approach						
27. I generate a Strengths-Weaknesses-Opportunities-Threats (SWOT) analysis in my decision making						
28. I present contingencies or achievable options as part of my decision making						
29. My decision making is transparent						
30. I understand the context of the decision I am being asked to make						
31. I understand the importance of the decisions I make						
32. I use a structured approach in my decision making						
33. I assign qualitative values to its decision-making criteria						
34. I assign quantitative values to its decision-making criteria						
35. I receive training in the science of decision making						
36. I use intuition or "gut-feeling" in my decision making						
37. My professional experience is important when having to make challenging decisions						
**B. Decision-making style**						
38. Emotion is part of my decision making						
39. I have experienced "paralysis by analysis" caused by my slow decision making						
40. I have experienced a negative outcome by a decision not being made						
41. In my decision making, I make the same mistakes as in the past						
42. Recent or dramatic events greatly impact my decision making						
43. My procrastination has resulted in a negative outcome						
44. My decision making could be improved by assigning relative importance to decision criteria						
45. I underestimate problems which adversely impact my decision making						
46. I continue with projects/products which should be terminated at an early stage						
47. I feel that I could make better quality decisions						

© 2017 M.S. Salek, S.R. Walker & R. Donelan
